# Quercetin protects islet β-cells from oxidation-induced apoptosis via Sirt3 in T2DM

**DOI:** 10.22038/ijbms.2021.52005.11792

**Published:** 2021-05

**Authors:** Jian-Yun Wang, Ya-Xing Nie, Bing-Zheng Dong, Zhi-Chen Cai, Xuan-Kai Zeng, Lei Du, Xia Zhu, Xiao-Xing Yin

**Affiliations:** 1 Jiangsu Key Laboratory of New Drug Research and Clinical Pharmacy, Xuzhou Medical University, Xuzhou 221002, Jiangsu, PR China; 2 Department of Urology, Xuzhou Central Hospital, The Affiliated School of Clinical Medicine of Xuzhou Medical University, Xuzhou 221009, Jiangsu, PR China; 3 Xuzhou Jiasheng Pharmaceutical Technology Co., Ltd., Xuzhou 221000, Jiangsu, PR China

**Keywords:** Apoptosis, β-cell, Oxidative stress, Quercetin, Sirt3

## Abstract

**Objective(s):**

Sirt3 may regulate ROS production and might be involved in β-cell apoptosis, which plays an important role in the progression of type 2 diabetes mellitus (T2DM). Quercetin is a potent anti-oxidative bioflavonoid, but its effects on T2DM remain to be explored. This study aimed to investigate the effects of quercetin on β-cell apoptosis and explore its mechanisms.

**Materials and Methods:**

The effects of quercetin were conducted on db/db mice and INS1 cells. Fasting blood glucose was determined by the colorimetric method, serum insulin was measured by enzyme-linked immunosorbent assay (ELISA). Meanwhile, Sirt3 in INS1 cells was knocked down by plasmid transfection. The antioxidant proteins (SOD2 and CAT), apoptosis proteins (cleaved Caspase-3, Bax, and BCL-2), and Sirt3 protein in pancreases and INS1 cells were determined by western blotting.

**Results:**

When INS1 cells and diabetic mice were treated with quercetin, the levels of SOD2, CAT, and Sirt3 proteins were increased, the levels of cleaved Caspase-3 and the ratio of Bax to BCL-2 were decreased at different degrees, along with reduced blood glucose levels and elevated insulin levels in diabetic mice. When Sirt3 was knocked down in INS1 cells, increase of two antioxidants and decrease of cell apoptosis generated by quercetin could not occur.

**Conclusion:**

Quercetin protected islet β-cells from oxidation-induced apoptosis via Sirt3 in T2DM, which would be beneficial to develop new strategies for preventing β-cell failure in T2DM.

## Introduction

Worldwide prevalence of type 2 diabetes mellitus (T2DM) has reached 8.5% among adults, and it is characterized by elevated glucose concentrations and failing insulin secretion (1). Increased apoptosis of islet β-cell is thought to be a key factor in impairing β-cell function by reducing the β-cell mass, which is occupying a dominant position in the pathophysiology of β-cell function failure (2, 3). Hyperglycemia is widely accepted as the feature of T2DM. However, *in vivo* and *in vitro* studies have revealed that acute or transient hyperglycemia could bring about the injury of islet β-cells. The damage of beta cells can further aggravate hyperglycemia and lead to further injury of beta cells, which was termed as “glucotoxicity” (2, 4-6). Therefore, high glucose is not only a consequence but also an important upstream factor.

 Although the etiology of the disease is not well defined, evidence suggests that oxidative stress or reactive oxygen species (ROS) induced by high glucose have a central role in the onset of T2DM, which can directly result in the injury of islet β- cells (7, 8). Sirt3, an NAD^+^-dependent protein deacetylase, often localized to mitochondria, is a member of the Sirtuin family of proteins (9). In many aging and metabolic diseases, it plays a major role in the oxidative stress response by deacetylating and modifying the enzymatic activities of several mitochondrial proteins (10, 11). Studies found that Sirt3 also directly up-regulates the expression of SOD2 and catalase (CAT) in mitochondria (10, 12). Caton *et al*. found that decreased Sirt3 contributes to the dysfunction of beta cells in T2DM by suppressing ROS production and exerting anti-inflammatory effects (13). All of these suggest that Sirt3 might be a key factor in the apoptosis of beta cells in T2DM due to its regulation of oxidative damage. 

Quercetin is a bioflavonoid compound detected in fruits and vegetables that shows multiple pharmacological effects, including reducing the risk of cardiovascular and kidney diseases, anti-tumor activity, and anti-oxidation, anti-virus, and anti-inflammatory effects (14, 15). In recent years, researchers paid much attention to the anti-oxidant activities of quercetin in many diseases, such as neurodegeneration and cardiovascular diseases (16-18). We previously found that quercetin had antidiabetic cataract effects by inhibition of oxidative stress and the polyol pathway (19). Research about quercetin for diabetes exists but it is mostly focused on diabetic complications, such as diabetic cardiomyopathy and diabetic nephropathy (20, 21). Few studies about quercetin were found in islet β-cells in T2DM and the underlying mechanism remains to be explored. 

In the present study, we investigated the effects of quercetin on the β-cell apoptosis under the high glucose condition in the* in vitro* and *in vivo* experiments and studied the effect of Sirt3 in cell apoptosis. Our research might provide strong support for application of quercetin in the prevention and treatment of T2DM. 

## Materials and Methods

***Materials***

Anti-Sirt3 (No.5490), anti-catalase (No. BS1616), anti-SOD2 (No. 13141), and anti- c-Caspase3 antibody (No.9665) were purchased from Cell Signaling Technology, Inc (Beverly, MA, USA). Anti-Bcl-2 (No. AF6139), anti-Bax (No. AF0120) antibodies were obtained from Affinity Technology, Inc. (USA). The mouse insulin kit (ER010-96) was bought from ExCell Bio Technology, Ltd (China). Quercetin (No. Q4951) was supplied by Sigma Technology, Inc. (USA), and the purity was more than 95% by HPLC. All of the secondary antibodies were purchased from the Beyotime Institute of Biotechnology (Nanjing, China). Other reagents were of analytical grade.

***Cell culture***

INS1 beta cells were donated by Professor Xiao Han at Nanjing Medical University. INS1 cells were cultured in RPMI 1640 medium containing 10% (vol./vol.) FBS, 10 mmol/l HEPES, 2 mmol/l L-glutamine, 1 mmol/l sodium pyruvate, and 50 μ mol/l β- mercaptoethanol. Cells were incubated at 37 °C in a humidified atmosphere of 5% CO_2_. Quercetin was dissolved in 0.1% DMSO with a final concentration.

***Transfection and groups***

During the experiments, cells were transfected with Sirt3-specific small hairpin RNA (shSirt3) or with scrambled shRNA control according to the manufacturer’s instructions. Sequences of Sirt3 shRNA oligonucleotides were as follows: 5’-acGGGCTGACGTGATGGCAGA-3’ and 5’- acGGGCTGACGTGATGGCAGA-3’ (Shanghai GeneChem Co., Ltd., Shanghai, China). Then the scrambled shRNA control cells were divided into three groups: one group was exposed in 11.1 mmol/ glucose (NG group), one group was exposed in 60 mmol/l glucose (HG group), and one group was treated with 60 mmol/l glucose and 20 µml/l Quercetin (HG+QE). The transfected ShSirt3 cells were also treated with 60 mmol/l glucose and 20 μml/l Quercetin (shSirt3+HG+QE). All cells were incubated for 48 hr before collection. 

***Experimental animals***

The animal procedures were performed in accordance with the Guiding Principles for Care and Use of Laboratory Animals of Xuzhou Medical University. The protocol was approved by the Committee on the Ethics of Laboratory Animals of Xuzhou Medical University. The 8-week old genetically diabetic C57BL/KSJ db/db mice and their age-matched nondiabetic littermates C57/KSJ db/m (db/m group, used as controls) were obtained from Nanjing General Hospital of Nanjing Military Command. The db/db mice were randomly divided into db/db group, db/db mice treated with a low dose of quercetin (QEL group, 50 mg/kg/d), and db/db mice treated with a high dose of quercetin (QEH group,100 mg/kg/d) via oral gavage. Quercetin was dissolved in 1% sodium carboxymethyl cellulose (CMC-Na) solution. And the control db/m mice were administered 1% CMC-Na solution of the same volume. Mice were housed in an animal facility conditioned with 12:12 hr of light-dark cycles and allowed free access to normal food and water. All mice were sacrificed after eight-week treatment via oral gavage when mice were 16 weeks old. Blood samples were collected for blood glucose and insulin determination. The pancreas was removed from each mouse for protein analysis.

***The determination of fasting blood glucose by colorimetric method***

Fasting blood glucose was measured using the glucose oxidase method with kits purchased from Rongsheng-Biotech Co. Ltd. (Shanghai, China) in accordance with the manufacturer’s instructions. The assay was based on the reaction of phenol and 4-aminoantipyrine with glucose to produce a red complex; and the absorbance was measured at 505 nm. 

***The levels of insulin by Enzyme***-***linked immunosorbent assay (ELISA)***

Insulin in the serum was first combined with mouse insulin monoclonal antibody before combination with streptavidin-horseradish peroxidase, the optical density of the colored immune complex was then measured at 450 nm. The levels of insulin were determined using a mouse insulin kit according to the manufacturer’s instructions.

***Western blotting analysis***

Total proteins of INS1 cells or pancreases were extracted and prepared as described (22). Briefly, the cells were lysed on ice with radioimmunoprecipitation assay (RIPA) buffer, protein concentrations were determined with the BCA protein assay kit (Beyotime Institute of Biotechnology). Then these proteins were separated by 8% SDS-PAGE electrophoresis, transferred to PVDF membranes, and blocked for 2 hr using 5% non-fat milk. Subsequently, blocked membranes were probed with primary antibodies overnight. Immunoblotted membranes were then incubated with corresponding secondary antibodies for 1 hr. The protein bands were detected using the Odyssey instrument (Gene Corporation, USA). The densities of signals on the blots were measured using the ImageJ2x software package.

***Statistical analysis***

Data were expressed as means±SD. Statistical analyses were performed using one-way analysis of variance (ANOVA) with Dunn’s Multiple Comparisons test for multiple data comparison. The *P*-values of less than 0.05 were considered significant (statistical significance: *P*<0.05 or *P*<0.01).

## Results

***The effects of quercetin on the levels of fasting blood glucose and serum insulin in T2DM mice***

**Figure 1:**
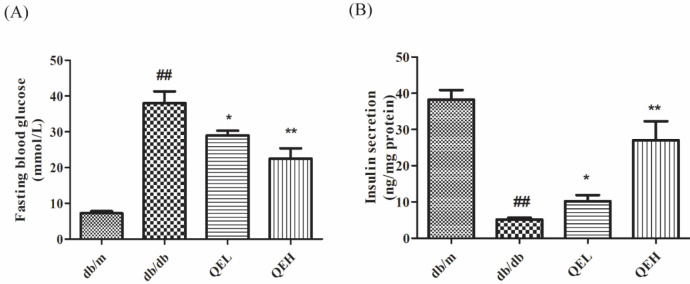
Quercetin decreased the levels of fasting blood glucose and increased the levels of serum insulin in T2DM mice

To observe the effects of quercetin in T2DM organisms, we detected the levels of fasting blood glucose and serum insulin in 16-week-old diabetic C57BL/KSJ db/db mice and their age-matched nondiabetic littermates db/m mice. As the results indicate in Figure 1, when compared with those in the db/m group, the level of fasting blood glucose in the db/db group was raised (*P*<0.01), suggesting that db/db mice suffered severe hyperglycemia. Meanwhile, the serum insulin level in the db/db group was significantly decreased (*P*<0.01), suggesting that the islet function of 16-week db/db mice had entered the failure stage and hyperglycemia was a probable result of islet beta-cell failure. When diabetic mice were administrated two doses of quercetin (QEL and QEH), we could find that the levels of fasting blood glucose in db/db mice were all decreased and serum insulin levels were all increased in contrast to those in the db/db group (*P*<0.01 or *P*<0.05). These results indicated that quercetin was an effective compound that could ameliorate blood glucose level and serum insulin secretion.

***The effects of quercetin on cell apoptosis in the pancreas of T2DM mice***

**Figure 2:**
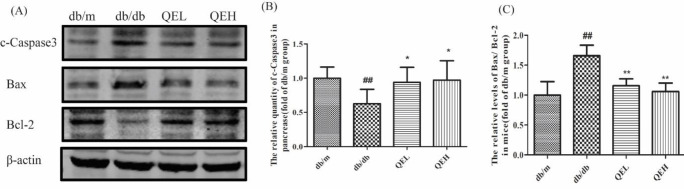
Quercetin inhibited cell apoptosis in the pancreas of T2DM mice

Since cell apoptosis plays an important role in the pathology of T2DM, we determined the changes of classical apoptosis indexes in pancreases by Western blotting: the apoptosis-promoting protein - Bax, the apoptosis suppression protein - Bcl-2, and the effector caspase protein - Caspase3 (Figure 2A). According to densitometric analysis in Figures 2B and 2C, the ratio of Bax to Bcl-2 protein (Bax/BCL-2) and the cleaved-Caspase3 (c-Caspase3, the activated Caspase3) in the db/db group were all elevated when compared with those in the db/m group (*P*<0.01), suggesting that cell apoptosis in diabetic pancreases were increased. After 8 weeks of treatment with different doses of quercetin, the levels of Bax/Bcl-2 proteins and the c-Caspase3 protein were reversed (*P*<0.05 or *P*<0.01), demonstrating that quercetin had an anti-apoptosis effect. 

***The effects of quercetin on expression of Sirt3 protein and anti-oxidants in the pancreas of T2DM mice***

**Figure 3:**
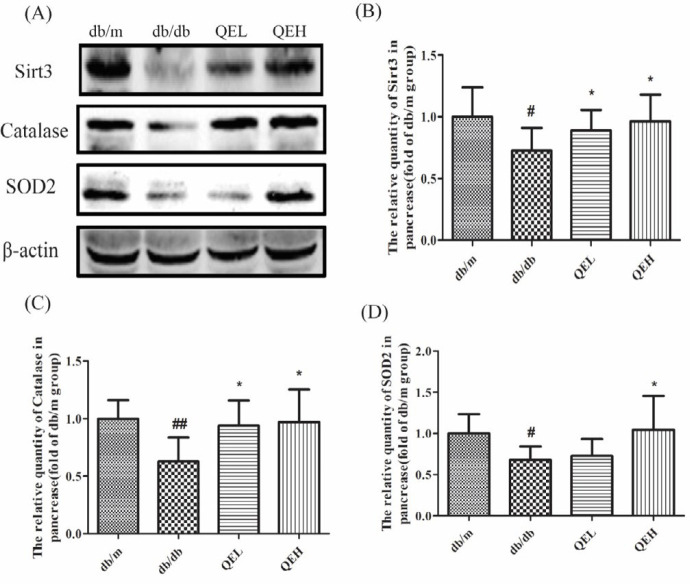
Quercetin increased the expression of Sirt3 protein and antioxidants in the pancreas of T2DM mice

Studies have revealed that Sirt3 plays a pivotal role in ROS clearance because of its effects on the activity or quantity of anti-oxidants, such as SOD2 and CAT, the quantity of Sirt3 proteins and the two anti-oxidants (SOD2 and CAT) in the pancreas were also determined by Western blotting (Figure 3). The results indicated that the quantities of Sirt3, SOD2 and CAT proteins were all significantly decreased in the db/db mice in contrast with the db/m mice (*P*<0.05 or* P*<0.01); while Sirt3 and CAT proteins were all improved in the QEL and QEH groups (*P*<0.05), and SOD2 protein was also increased in the QEH group (*P*<0.05) when compared with those in the db/db group. These results disclosed that quercetin played its strong anti-oxidation role, and this effect was probably related to its regulation on Sirt3 expression in pancreases in T2DM.

***Quercetin increased the anti-oxidant expression of β-cells by elevation of Sirt3 expression in the in vitro experiment***

**Figure 4:**
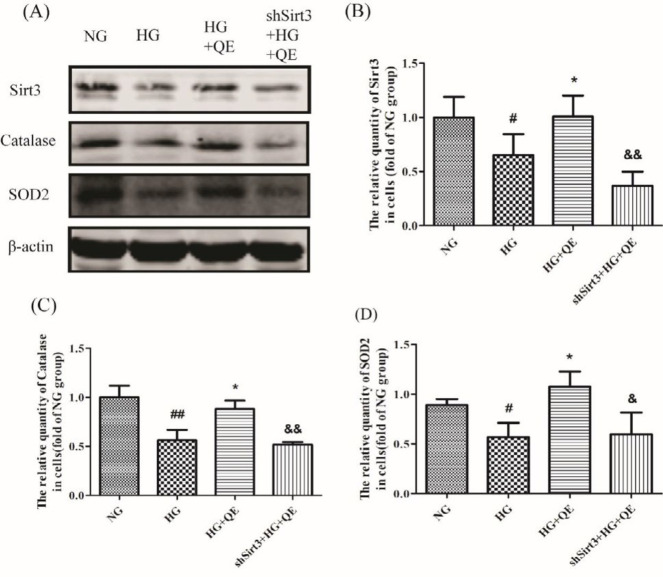
Quercetin increased the antioxidant expression of β-cells by elevation of Sirt3 expression in INS1cells

To further ascertain the effects of quercetin on T2DM and its mechanism, a cell line special for the function of pancreatic β-cell, INS1 cell was used here. Meanwhile, Sirt3 was knocked down by transfecting shSirt3 into the INS1 cell to clarify the role of Sirt3 in the anti-oxidation of quercetin. As was shown in Figure 4, a lower quantity of Sirt3, SOD, and CAT proteins by Western blotting were produced in the HG group in contrast to the NG group (*P*<0.05 or* P*<0.01), suggesting that high glucose was an important factor resulting in the decrease of three indexes. However, when quercetin was added in, their quantities were strikingly elevated (HG+QE group) in contrast to the HG group (*P*<0.05). When Sirt3 was knocked down in INS1 cells, the increase of two anti-oxidants produced by quercetin in high glucose (shSirt3+HG+QE group) could not be seen (Figures 4C and 4D) in contrast to the HG+QE group (*P*<0.05 or *P*<0.01). These results told us that, once the Sirt3 protein could not be increased, the increase of CAT and SOD2 proteins induced by quercetin would not occur. That was to say, it was just by Sirt3 that quercetin promoted CAT and SOD2 production under the high glucose condition. 

***Quercetin reduces the apoptosis of*** ***β*** ***-*** ***cells by elevation of Sirt3 expression in the in vitro experiment***

**Figure 5:**
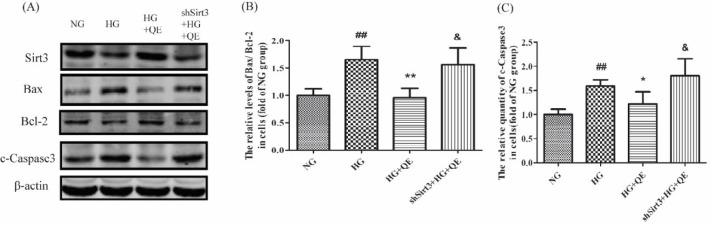
Quercetin reduces apoptosis of β-cells by elevation of Sirt3 expression in INS1 cells

Finally, the apoptosis in INS1 cells was determined by Western blotting. As was shown in Figure 5, when compared with the NG group, the levels of Bax/Bcl-2 and c-Caspase3 protein in the HG group were augmented (*P*<0.01), suggesting that high glucose was an important factor in increasing β-cell apoptosis. When quercetin was added into the high glucose, reverse results were found in the HG+QE group in contrast to the HG group, indicating that quercetin could protect β-cells against apoptosis from high glucose. When Sirt3 was knocked down, the decrease of beta-cell apoptosis produced by quercetin in the shSirt3+HG+QE group could not be seen, in contrast to the HG+QE group (*P*<0.05). These results told us that, once the Sirt3 protein could not be increased, the anti-apoptosis function of quercetin would not conduct. That is to say, it was just via Sirt3 that quercetin prevented the apoptosis of islet β-cells from the high glucose.

## Discussion

Diabetes is a chronic disease characterized by disordered metabolism and abnormally high blood glucose levels. Studies have revealed that β-cell apoptosis is increased in rodents and patients with T2DM (3, 23). Therefore, research about clarifying the mechanism of β-cell apoptosis in T2DM and the method of preventing β-cell apoptosis draws much attention. 

Quercetin is a polyphenolic flavonoid compound. It is abundantly present in kales, onions, berries, apples, red grapes, broccoli, and cherries, as well as tea and red wine (18). Modern studies have shown that quercetin prevents various diseases, such as osteoporosis, tumors, neurodegeneration, and cardiovascular diseases (14, 24, 25). We previously have found that quercetin prevented renal fibrosis and cataracts in T2DM (19, 21). As for apoptosis, quercetin was found to play a different role. It was found to induce apoptosis in many kinds of cancerous cells, such as breast cancer cells, myeloid leukemia cells, ovarian cancer cells, and so on (26-28). In recent years, more and more researchers have found quercetin has an anti-apoptotic potential, which has been studied in lots of normal cells, e.g. chondrocytes and pulmonary arterial smooth muscle cells (29, 30). The different effects of quercetin on cell apoptosis might be related to the cell type and the concentration of quercetin (31). Though the mechanism of β-cell failure in T2DM remains unclear, chronic oxidative stress induced by glucotoxicity is thought to be a major factor responsible for the apoptosis of β-cell (32, 33). Glucose can be converted to enediol when intracellular concentrations exceed the glycolytic capacity of β-cells, which leads to superoxide formation (34). Studies reveal that β-cells are more susceptible to oxidative damage than other cells because β-cells contain lower levels of anti-oxidant enzymes, such as CAT, glutathione peroxidase, and mitochondrial manganese superoxide (35, 36). In the present study, we investigated the effects of quercetin on T2DM and explored the mechanism of its anti-oxidant effect. We found that two anti-oxidant proteins (SOD2 and CAT) were decreased in 16-week-old diabetic mice, along with severe hyperglycemia and low insulin levels. Meanwhile, we found that cell apoptosis in diabetic pancreases was increased, through detection of cleaved Caspase-3 protein and mitochondrial apoptosis protein Bax/BCL-2. Our results implied that the oxidative and anti-oxidant system was out of balance. β-cell apoptosis and the consequent hyperglycemia were probable results of oxidative stress. After 8-week treatment of quercetin, all indexes were reversed at different degrees, especially in the high dose of quercetin group. The *in vitro *experiment also indicated that high glucose induced the apoptosis of β-cells and decreased the quantity of two anti-oxidant proteins. The addition of quercetin into INS1 cells inhibited oxidative stress and cell apoptosis. All of our results showed that quercetin was a potent anti-oxidative and anti-apoptotic compound. Some studies reveal that quercetin is even a stronger anti-oxidant than vitamins C and E (18, 37). Our result was similar to those of Alam *et al*., who report that quercetin ameliorates hyperglycemia through inhibiting oxidative stress in alloxan-induced type 2 diabetic mice (38). Studies also found that quercetin is a signiﬁcant anti-diabetic flavonoid in hypoglycemic effect, insulin resistance, and diabetic complications (39-41). Therefore, quercetin is a promising flavonoid for the prevention and treatment of diabetes. However, the mechanism of quercetin against β-cell apoptosis needs further investigation. 

Sirt3, the mitochondrial NAD^+^-dependent deacetylase, widely expressed in liver, fat, and beta cells of the pancreas, may regulate mitochondrial function and biosynthetic pathways such as glucose and fatty acid metabolism and the tricarboxylic acid (TCA) cycle, oxidative stress, and apoptosis by reversible protein lysine deacetylation (42). In the present study, we found that Sirt3 protein was decreased in 16-week db/db mice and in INS1 cells exposed to high glucose, while treatment with quercetin improved Sirt3 expression. Since Sirt3 was involved in production of ROS and oxidative stress(43), was the beneficial function of quercetin on cell apoptosis ascribed to its regulation on Sirt3? Given that, Sirt3 was knocked down in INS1 cells in normal glucose, then the Sirt3 knocked-down cells were exposed to high glucose and quercetin (shSirt3+HG +QE). These results showed us that, once the Sirt3 protein could not be increased, the increase of two anti-oxidants (CAT and SOD2) and decrease of cell apoptosis generated by quercetin would not occur. That is to say, it was just by Sirt3 that quercetin promoted CAT and SOD2 production and subsequently prevented β-cell apoptosis in high glucose. 

Therefore, in the present study, we found that quercetin increased the expression of Sirt3, augmented the subsequent anti-oxidant (CAT and SOD2) production, and finally inhibited the β-cell apoptosis. In other words, quercetin protected islet β-cells from oxidation-induced apoptosis via Sirt3 in T2DM. Consequently, Sirt3 is a crucial factor in the protection of β-cells by quercetin. Studies found that Sirt3 increases the anti-oxidant capacity of cells by deacetylating mitochondrial proteins including SOD2 in age-related diseases such as neurodegenerative diseases and cardiovascular diseases (44, 45). We interestingly found that quercetin has an effect on the quantities of SOD2 and CAT by up-regulation of Sirt3 in β-cells in T2DM. The mechanism still remains unclear. Similar studies about Sirt3 regulating the quantity of anti-oxidants were also found in arterial thrombosis, traumatic brain injury, ischemic heart diseases, and so on (12, 46, 47). Research reported that Sirt3 protected cells against oxidative stress by positively regulating anti-oxidant enzymes (SOD2 and CAT) via increasing expression of FoxO3a in the nucleus (47, 48) because SOD2 and CAT were the target genes of FoxO3a. This mechanism via targeted Sirt3-FoxO3a might also be involved in the regulation of quercetin on anti-oxidant enzyme expression and the consequent β-cell protection, which needs further investigation. Some studies found that quercetin alleviated the H_2_O_2_-induced reduction in cell viability and improved β-cell functionality via stimulation of ERK1/2 phosphorylation and inhibition of H_2_O_2_-induced p38 MAPK phosphorylation (49, 50). Accordingly, our results provided a new mechanism for the protection of quercetin on oxidation-induced β-cell apoptosis in T2DM.

(A) Levels of fasting blood glucose in mice; (B) Levels of serum insulin in mice. Data were presented as the mean±SD (n=6). Differences between groups were evaluated by one-way ANOVA. QEL and QEH represent db/db mice treated with 50 mg/kg/d quercetin and 100 mg/kg/d quercetin, respectively. ^##^*P*<0.01, compared with the db/m group; ^*^P<0.05, ^**^P<0.01, compared with the db/db group

T2DM: type 2 diabetes mellitus

(A) Bands of c-Caspase3, Bax, and Bcl-2 by western blotting in the pancreas of mice. (B) Densitometric analysis of c-Caspase3 in the bands. (C) Ratio of Bax to Bcl-2 (Bax/Bcl-2) by densitometric analysis in the bands. β-actin was used as the endogenous control, and the relative quantity of protein was normalized to that of the db/m group. QEL and QEH represent db/db mice treated with 50 mg/kg/d quercetin and 100 mg/kg/d quercetin, respectively. Data were presented as the mean±SD (n=6). Differences between the groups were evaluated by one-way ANOVA. ^##^*P*<0.01, compared with the db/m group; ^*^P<0.05, **P<0.01, compared with the db/db group

T2DM: type 2 diabetes mellitus

(A) Bands of Sirt3, catalase, and SOD2 by Western blotting in the pancreas of mice. (B-D) Densitometric analysis of Sirt3, catalase, and SOD2 in the bands of the pancreas. β-actin was used as the endogenous control, and the relative quantity of protein was normalized to that of the db/m group. QEL and QEH represent db/db mice treated with 50 mg/kg/d quercetin and 100 mg/kg/d quercetin, respectively. Data were presented as the mean±SD (n=6). Differences between the groups were evaluated by one-way ANOVA. ^#^*P*<0.05, ^##^*P*<0.01, compared with the db/m group; *P<0.05, compared with the db/db group

T2DM: type 2 diabetes mellitus

(A) Bands of Sirt3, catalase and SOD2 by western blotting in INS1 cells. (B-D) Densitometric analysis of Sirt3, catalase, and SOD2 in the bands of cells. β-actin was used as the endogenous control, and the relative quantity of protein was normalized to that of the NG group. Cells were divided into NG group (11.1 mmol/ glucose), HG group (60 mmol/lglucose), HG+QE group (60 mmol/l glucose and 20 µml/l Quercetin), and shSirt3+HG+QE group (transfected ShSirt3 cells exposed to 60 mmol/l glucose and 20 μml/l Quercetin). Data were presented as the mean±SD (n=4). Differences between the groups were evaluated by one-way ANOVA. ^#^*P*<0.05, ^##^*P*<0.01, compared with the NG group; ^*^P<0.05, compared with the HG group; ^&^*P*<0.05, ^&&^*P*<0.01, compared with the HG+QE group

(A) Bands of Sirt3, C-Caspase3, Bax, and Bcl-2 by western blotting in INS1 cells

(B) Ratio of Bax to Bcl-2 (Bax/Bcl-2) by densitometric analysis in the bands. (C) Densitometric analysis of c-Caspase3 in the bands. β-actin was used as the endogenous control, and the relative quantity of protein was normalized to that of the NG group. The cells were divided into NG group (11.1 mmol/ glucose), HG group (60 mmol/l glucose), HG+QE group (60 mmol/l glucose and 20 µml/l Quercetin), and shSirt3+HG+QE group (transfected ShSirt3 cells exposed to 60 mmol/l glucose and 20 μml/l Quercetin). Data were presented as the mean±SD (n=4). Differences between the groups were evaluated by one-way ANOVA. ^##^*P*<0.01, compared with the NG group; ^*^P<0.05,^ **^P<0.01, compared with the HG group; ^&^*P*<0.05, compared with the HG+QE group

## Conclusion

This study found that quercetin protected islet β-cells from oxidation-induced apoptosis via Sirt3 in T2DM. We reported the first results concerning the role of Sirt3 in β-cell protection by quercetin under the high glucose condition. Our results would be beneficial to develop new strategies for preventing islet β-cell failure in T2DM.
